# Spatial heterogeneity of malaria vectors and malaria transmission risk estimated using odour-baited mosquito traps

**DOI:** 10.1186/1475-2875-13-S1-P41

**Published:** 2014-09-22

**Authors:** Alexandra Hiscox, Tobias Homan, Corné Vreugdenhil, Bruno Otieno, Anthony Kibet, Collins K Mweresa, Ron van Lammeren, Wolfgang R Mukabana, Willem Takken

**Affiliations:** 1 Laboratory of Entomology, Wageningen University and Research Centre, Wageningen, The Netherlands; 2 Laboratory of Geo-information Science and Remote Sensing, Wageningen University and Research Centre, Wageningen, The Netherlands; 3 International Centre of Insect Physiology and Ecology, Nairobi, Kenya

## Background

Prior to the commencement of a large-scale malaria intervention study on Rusinga Island, western Kenya, intensive baseline surveillance of the mosquito population was performed using odour-baited traps. The survey aimed to determine the relative abundance and species composition of malaria vectors, and to measure seasonal and spatial heterogeneity in populations. Human malaria prevalence was combined with entomological data to provide information about malaria transmission risk before the intervention began.

## Materials and methods

From September 2012 until June 2013, mosquito monitoring took place over successive six-week sampling periods. MM-X traps baited with attractant lures and carbon dioxide were used to collect mosquitoes from inside and outside houses, and a new random sample of 80 households was drawn for each sampling round. During the baseline period, malaria prevalence was measured twice in a randomly selected 10% of the human population. A QuickBird satellite image and digital elevation map were used to describe environmental features of the island. Mosquitoes were initially identified on the basis of morphology and anophelines were processed by PCR to confirm species identifications.

## Results

Odour-baited MM-X traps proved to be a good tool for monitoring malaria vectors inside and outside houses. Using this tool a marked temporal and spatial heterogeneity was described for the malaria vector species *Anopheles gambiae s.s*., *An. arabiensis* and *An. funestus*. Regions of potentially high malaria transmission intensity were identified after mapping the distributions of malaria mosquitoes and Plasmodium-positive persons. Despite studying a range of environmental and topographical features, no strong associations were found between environmental variables and the presence or absence of adult Anopheles.

## Conclusions

Malaria vectors and malaria prevalence are not homogeneously distributed across Rusinga Island; the risk of malaria transmission is therefore greater in some areas than others. The finding that environmental features were not closely associated with adult malaria vector distribution, indicates that other factors, such as house construction or the presence of livestock, may play a more important role in the decision of a female anopheline to approach the domestic environment of a particular house in search of a blood meal. The findings of this study demonstrate how trans-disciplinary data can be integrated to provide a better understanding of mosquito population dynamics and malaria transmission risk. Intensive mosquito monitoring before the commencement of, as well as during, a large-scale malaria intervention study, contributes valuable information which will be used in describing the eventual impact of the intervention.

